# Cytotoxic and Antioxidant Effects of Phoenix dactylifera L. (Ajwa Date Extract) on Oral Squamous Cell Carcinoma Cell Line

**DOI:** 10.1155/2022/5792830

**Published:** 2022-02-07

**Authors:** Khushboo Shahbaz, Jawaad Ahmed Asif, Tang Liszen, Asma Abdullah Nurul, Mohammad Khursheed Alam

**Affiliations:** ^1^Oral Medicine Department, School of Dental Sciences, Universiti Sains Malaysia, Kota Bharu, Kelantan, Malaysia; ^2^Oral and Maxillofacial Surgeon, Prince Mutaib Bin Abdulaziz Hospital, Sakakah, Al-Jouf, Saudi Arabia; ^3^Maxillofacial Surgery Unit, School of Dental Sciences, Universiti Sains Malaysia, Kota Bharu, Kelantan, Malaysia; ^4^School of Health Sciences, Universiti Sains Malaysia, Kota Bharu, Kelantan, Malaysia; ^5^Orthodontics, Preventive Dentistry Department, College of Dentistry, Jouf University, Saudi Arabia; ^6^Center for Transdisciplinary Research (CFTR), Saveetha Dental College, Saveetha Institute of Medical and Technical Sciences, Saveetha University, Chennai, India; ^7^Department of Public Health, Faculty of Allied Health Sciences, Daffodil International University, Dhaka, Bangladesh

## Abstract

**Aim:**

The aim of the current study is to investigate the antioxidant and apoptotic potential of Ajwa date flesh (ADF) and Ajwa date pit (ADP) extract on human squamous cell carcinoma cell line (HSC-2).

**Method:**

ADF and ADP were extracted with a solvent extraction method using hexane, acetone, and ethanol, which were then subjected to antioxidant assay by 2,2-diphenyl-1-picrylhydrazyl (DPPH). HSC-2 cells were then treated with different concentrations of ADF and ADP extract for 24, 48, and 72 hours. MTT assay was performed to assess the antiproliferative effect, and Annexin V-FITC was used for the detection of cellular apoptosis.

**Results:**

Acetone extracts of ADF and ADP had the highest radical scavenging and antioxidant activities followed by the ethanolic extracts, whereas ADP appeared to have significantly higher antioxidant effects than ADF. MTT assay demonstrated that acetone extracts of ADF and ADP were significantly cytotoxic against HSC-2 cells in a dose- and time-dependent manner. The half inhibitory concentration (IC50) of ADF was found to be 8.69 mg/ml at 24 h, and the maximum cell growth inhibition was observed at 50 mg/ml. The IC50 for the ADP was found to be 0.97 mg/ml at 24 h, and the maximum cell growth inhibition was observed at 5 mg/ml. Statistical analysis of the flow cytometry assay showed that the treatment with ADF and ADP extracts had a significant apoptotic effect which occurred in a dose-dependent manner. HSC-2 cells were seen in the late apoptotic stage with higher doses of ADF and ADP extract. ADP extract demonstrated higher apoptotic activity than ADF extract. In addition, combined treatment of ADF and ADP was also performed on HSC-2 cells which demonstrated higher apoptotic activity when compared to the single extract.

**Conclusion:**

Ajwa date fruit has a promising cytotoxic effect by inhibiting the growth and proliferation of OSCC cells and inducing cell death by apoptosis.

## 1. Introduction

Oral squamous cell carcinoma (OSCC) is the 6^th^ most common cancer around the world. It has been documented that 90% of all oral malignancies are squamous cell carcinoma (SCC) [1]. With an increase in the prevalence and mortality rate, there were an estimated 377,713 new cases and 177,757 deaths from oral cancer in 2020 [2]. The incidence of the disease is influenced by several factors such as use of tobacco, betel quid chewing, alcohol drinking, infection with HPV, genetics, radiation, unhealthy diet, and physical inactivity [3]. The 5-year survival rate is 50%, but if detected in early stages, it is increased to 60-80% [4]. Despite advancement in surgical approach and radiation therapy, the adverse effects of these treatments include anaemia, loss of appetite, and peripheral neuropathy leading to poor quality of life. Therefore, there is a need to develop an effective treatment with potent anticancer activity and less adverse effects [5].

A new approach in the management of oral cancer treatment is by antioxidant therapy. Reduction in oxidative stress which damages the intracellular structure and DNA of cell can be achieved by antioxidants, which are responsible for the removal of free radicals. Studies have found that date fruit scavenges the free radicals and prevents the occurrence of macromolecular changes in living systems [6]. Studies have also found that Ajwa date flesh and pit have shown gastroprotective [7], hepatoprotective [8], antidiabetic [9], and anti-inflammatory properties [10] due to their strong antioxidant, antimutagenic, and anticancerous activity which can be attributed due to the presence of phenolic compounds and flavonoid glycosides [11] [12]. Ajwa date flesh and pit are also attributed for antimicrobial [13], antifungal [14], and antiviral activities [15]. In the religion of Islam, Ajwa dates have remarkable status as narrated in Hadith by Sahih Al-Bukhari: The Prophet (peace be upon him) said “Whoever eats seven Ajwa dates every morning will not be harmed by poison or witchcraft all that day until night comes” [16].

Ajwa are soft fleshy dates with blackish-brown colour. The edible part is high in sugar content and moisture whereas the pit part is high in crude protein [16], while both parts contain abundant number of dietary fibres and high amount of essential minerals which are necessary for the skeletal growth and maintenance of cellular functions in human body in contrast with other date varieties [12, 16–18]. Provitamin A, C, D, E, and K; riboflavin (B2); pyridoxine (B6); and niacin (B3) were also found in Ajwa date flesh [19–21]. Date fruits also contain sulphated-flavanol glycosides. This form of flavanols was not detected before in any fruit as well as vegetables [22, 23].

Ajwa date fruit reported to have high amount of carotenoids like beta-carotene and lutein and polyphenols like quercetin, isoquercetin, luteolin, apigenin, rutin, and anthocyanins when compared to other varieties [24]. These phytochemicals are known for their antioxidant property which help in the prevention and treatment of cancer through inhibition of cell cycle progression and induction of apoptosis by modulating signalling pathways which can regulate intracellular reactive oxygen species (ROS), Bcl-2/Bax, and p53/p21 pathways [25, 26].

The anticancerous mechanism was investigated in a study on human breast adenocarcinoma (MCF7) cell line with methanolic extract of Ajwa date. The result showed significant inhibition of cell proliferation in a dose- and time-dependent manner. Flow cytometric analysis showed that the cell death was due to apoptosis [27]. In another study, date seed oil also has shown chemoprotective effects and can be used in the prevention of skin cancer caused by UV radiation [28, 29]. Few other *in vitro* studies showed similar results of cytotoxic properties of Ajwa date extract with aqueous acetone on cervical (HeLa) cell line [30], Ajwa date extract with ethanol on hepatocellular carcinoma (HepG2) cell line [31], and lung cancer (NCI-H460) cell line [32].

The extraction of different phytochemical compounds is influenced by the polarity of the solvent. Anthocyanins are best extracted by ethanol [33], whereas acetone was the best solvent for the extraction of phenols and flavonoids [34]. Since polyphenols, flavonoids, and flavones attributed to the Ajwa date antioxidants and tissue protective properties, it is important to select a single solvent which can yield the maximum antioxidant from the fruit [35]. In the previous literature, different solvents have been used to extract different bioactive components according to their research needs; therefore, three solvents were selected in the present study to extract the maximum amount of polyphenols and flavonoids. To this date and to our best knowledge, no study has been conducted to oversee the cytotoxic and apoptotic effects of Ajwa date flesh and pit on human oral squamous cell carcinoma cell line (HSC-2).

## 2. Materials and Methods

### 2.1. Preparation of Ajwa Date

Ajwa dates are only cultivated in the outskirts of city of Madinah Al Munawwarah. Four kg of Ajwa dates was purchased from the “Tamar Market” of Al-Madinah Al Munawwarah, Kingdom of Saudi Arabia. Medium-sized Ajwa dates without any visible physical damage were selected and washed, first with tap water and then with distilled water three times under sterile environment to remove all the dust and soil particles; then dried with sterile cotton cloth; and air-dried under shade for one night. Ajwa dates were then manually pitted, and the seeds were washed again to remove any remaining date flesh and air-dried for another night. Ajwa date flesh was chopped to small pieces approximately 1 cm each using a surgical blade no. 10. Ajwa pits and the cut date flesh were then further dried completely using a freeze-drying method for a week separately in order to stabilize the samples and prevent microbial spoilage and hydrolytic rancidity [36]. Date pits were milled using a laboratory milling grinder with 30~300-micron finesse. The flesh part was crushed coarsely using a pestle and mortar. The powdered pits and flesh were weighed and made into aliquots of 50 g and sealed into airtight plastic bags stored at -40°C in dark until required for extraction.

### 2.2. Extraction of Date Flesh and Seed

In this study, different solvents such as n-hexane, acetone/H_2_O (70 : 30, *v*/*v*), and ethanol (70 : 30, *v*/*v*) were used successively in order to isolate a wide range of antioxidant compounds present in Ajwa date's flesh and pits. In this study, the order in which extraction was performed was n-hexane, acetone 70%, and ethanol 70%. Ajwa date's flesh and pit extractions were performed separately following the protocol described previously by [27, 37] with slight modifications.

A portion of freeze-dried contents (15 g) was extracted in n-hexane (150 ml) with a ratio of 1 : 10 (weight to volume) for 48 hours at room temperature in a flat bottom flask on a shaking incubator. Following extraction, the resultant extract was filtered using Whatman No. 1 filter paper. The remaining residue was further extracted in acetone/H_2_O (70 : 30) for 48 hours at room temperature with continuous agitation. The solution was filtered and evaporated under vacuum using a concentrator to give a dark brownish extract. Remaining insoluble residue was subjected to ethanol/H_2_O (70 : 30) extraction for 48 h at room temperature. The solution was filtered, and the filtrate was evaporated under vacuum to give dark brown concentrate. All the crude extracts were frozen, and the H_2_O removed by freeze-drying to yield a brown solid. All the extracts of Ajwa date's flesh and pits were weighed, sealed, labelled, and stored at -20°C in 50 ml tubes for analytical purposes.

### 2.3. Antioxidant Properties of Date Extract

#### 2.3.1. DPPH Assay

To evaluate the antioxidant capacity of Ajwa date's flesh and seed extracts from three different solvents, diphenyl picrylhydrazyl (DPPH) assay was carried out to obtain the highest biologically active extract according to the previously described protocol [38]. The working solution of DPPH in methanol was prepared daily for the measurement of antioxidants in the extracts using a UV spectrophotometer. To prepare the 0.1 mM of DPPH (molecular weight 394.32 g/mol) solution, 3.94 mg of DPPH was dissolved in 100 ml of methanol in a flask which was covered by aluminium foil. Three ml of this solution was then mixed with 100 *μ*l of various concentrations of sample extract solution in disposable microcuvettes. The samples were kept in a dark place for 30 min at room temperature before being measured for absorption at 517 nm using the spectrophotometer. A blank sample containing 3 ml of DPPH solution was measured daily to obtain an absorbance of 0.0 ± 0.02 units at 517 nm. Ascorbic acid was used as a standard reference for the comparison of results. A standard curve was prepared for calibration using six concentrations of ascorbic acid ranging from 1.5 to 50 mg/ml. The total antioxidants are expressed as milligrams per milliliter of ascorbic acid. The experiment was run in triplicate, and average was taken to calculate the radical scavenging activity using the following formula:

(1) $$\begin{array}{c} \%\text{inhibition}=\left[\frac{\left(A_{\text{b}}-A_{\text{s}}\right)}{A_{\text{b}}}\right]\times 100, \end{array}$$

where *A*_b_ is absorbance of control and *A*_s_ is absorbance of the sample.

### 2.4. Cytotoxic Properties of the Date Extract

#### 2.4.1. HSC-2 Cell Culture

HSC-2, RCB-1945, is a human oral squamous carcinoma cell line, which was purchased from Riken Cell Bank, Japan. HSC-2 cells were cultured in minimum essential medium (MEM) containing 2 mM L-glutamine, 10% fetal bovine serum (FBS), and 1% penicillin/streptomycin. The cells were incubated in 5% CO_2_ at 37°C incubation in a humidified CO_2_ incubator. Cells were maintained as a monolayer, and cell passage was performed every 3^rd^ or 4^th^ day.

### 2.5. Evaluation of Morphological Changes of HSC-2 Cells

Cells were cultured in 6-wells (10 mm) cell culture petri dishes at a seeding density of 2 × 10^5^ cells/well. After 24-hour attachment, the cells were treated with different concentrations of Ajwa date's flesh and pit extracts separately and in combination, whereas untreated cells served as control. The morphological changes were observed under an inverted microscope, and images were taken for the comparison with the untreated cell images at 48-hour treatment.

### 2.6. Assessing Cell Viability by MTT Assay

In this study, the 3-(4,5-dimethylthiazol-2-yl)-2,5-diphenyltetrazolium bromide (MTT) assay was used to determine the cytotoxic effect of Ajwa date's flesh and pit extracts on oral squamous cell carcinoma. A range of concentrations (0.8, 1.5, 3.1, 6.3, 12.5, 25, and 50 mg/ml) of ADF extract [27, 31, 39] and (0.08, 0.31, 0.63, 1.15, 1.25, 2.5, and 5 mg/ml) of ADP extract [40, 41] were selected with 70% aq. acetone.

The HSC-2 cells were harvested and seeded in a 96-well plate at a cell density of 5 × 10^3^ cells/well. Cell's attachment was confirmed under a microscope after 24 hours; the cells were then treated with different concentrations of ADF and ADP extracts separately. Control wells were treated with the same amount of complete growth media only. For every treatment and untreated control group, complete growth media without cells were added as a blank to reduce the background absorbance values. For every concentration of ADF and ADP extracts, 6 wells were used, three with treatment and three as blanks. All the experiments were carried out three times independently with 3 replicates in each experiment.

The cells were grown with sample extracts up to three different time points (24 h, 48 h, and 72 h) after which MTT assay was performed by removing the medium gently and adding 10 *μ*l of MTT solution with a final concentration of 5 mg/ml per well, and incubated at 37°C for about 4 hours until the purple crystals were formed. After that, the MTT solution was discarded from every well and 100 *μ*l of DMSO was added to dissolve the crystals. The 96-well plate was mounted on a microplate shaker and shaken for 15 minutes until the crystals completely dissolved. The absorbance value for each well was determined at an optical density at 570 nm wavelength using an ELISA microplate reader [42]. The 50% inhibitory concentration (IC_50_) of the ADF and ADP extract was also calculated at 24 h.

The cell viability (CV) percentage after treatment with ADF and ADP extract was calculated with the formula below:

(2) $$\begin{array}{c} \text{CV}\left(\%\right)=\frac{\text{absorbance of treatment cells}‐\text{absorbance of blanks}}{\text{absorbance of control cell}‐\text{absorbance of blanks}}\times 100. \end{array}$$

### 2.7. Detection of Apoptosis by Flow Cytometry Assay

Annexin V-FITC (fluorescein isothiocyanate) Apoptosis Detection Kit I was used to quantify apoptotic cells by flow cytometry following the manufacturer's instructions. For negative control, untreated cells were used. Briefly, HSC-2 cells at 2 × 10^5^ cells/ml density were incubated for 24 h to allow adherence of cells to the 6-well culture plate. After 24 hours, the cells were incubated with 8.69 mg/ml (IC_50_) and 25 mg/ml of ADF extract and 0.97 mg/ml (IC_50_) and 2.5 mg/ml of ADP extracts for 24, 48, and 72 hours. After the given time point, the cells were harvested by washing with PBS and trypsinizing with 400-450 *μ*l of trypsin. Cells were then centrifuged with 1 ml of cold PBS twice and once with 100 *μ*l of 1X binding buffer solution for 5 minutes each time at 157 × g. The cells were stained with 5 *μ*l Annexin V-FITC and 5 *μ*l Propidium Iodide (PI) for 15 min at 25°C in the dark and resuspended in 400 *μ*l of 1X binding buffer solution and immediately analysed by BD Accuri ™ C6 flow cytometry in 5 ml round-bottom polystyrene FACS tubes. Wavelengths of 533 nm and 585 nm were used for Annexin V-FITC and PI, respectively. 10,000 events per sample were recorded on forward scatter versus side scatter plot using BD Accuri™ C6 software. Control samples were prepared separately for the purpose of recording the HSC-2 cells according to their granularity and size. Cells with PI-only stain represent necrosis, PI and Annexin-V represent late apoptosis, cells stained with Annexin-V only represent early apoptosis, and unstained cells were evaluated as viable healthy cells.

### 2.8. Combination Treatment

A combination treatment of both ADF and ADP extracts was performed together to analyse if there was any synergistic effect in apoptosis of the cells.

IC_50_ concentrations from the MTT assay of ADF and ADP were combined together (8.69 mg/ml + 0.97 mg/ml) as a single treatment, and the results were recorded.

Likewise, next higher concentrations were combined for both the extracts (25 mg/ml + 2.5 mg/ml) and used as a single treatment to observe if it had significantly higher apoptotic effect than if used separately.

### 2.9. Statistical Analysis

The statistical analysis was carried out using SPSS 24 version. To compare the difference between the control and the treated groups for DPPH, cell proliferation and apoptosis assay. One-way ANOVA was conducted followed by either Tukey's post hoc or Dunnett's multiple comparison test. The IC_50_ values for DPPH and MTT assay were calculated using nonlinear regression analysis function with Prism GraphPad software (version 7). All experimental data was represented as the mean ± standard deviation (SD) of three independent experiments which were performed in triplicate. A *p* value of less than 0.05 (*p* < 0.05) was considered statistically significant.

## 3. Results

### 3.1. DPPH Assay

Assessment of the total antioxidant contents of the Ajwa date flesh's extract was performed. The assay could not be performed with n-hexane solvent due to the insufficient amount of extracted residue present to make a working solution to perform the assay. Therefore, antioxidant activity from the acetone/H_2_O (70 : 30, *v*/*v*) and ethanol/H_2_O (70 : 30, *v*/*v*) was studied.

**Figure 1:**
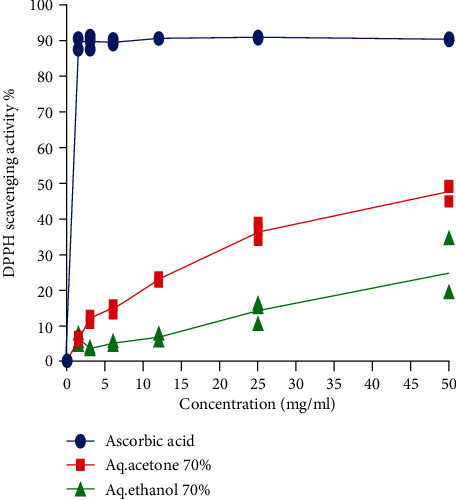
DPPH scavenging activity of different solvents of ADF extract at different concentrations. The curve for ascorbic acid has been used as a standard. Each value represents the mean of three independent experiments (*n* = 3).

The results of ADF sample of aq. acetone 70% and aq. ethanol 70% of 1 to 50 mg/ml concentrations are shown in Figure 1. Data shows that the percentage of inhibition of aq. acetone 70% ADF extract was higher than that of the aq. ethanol 70% ADF extract. The curve for ascorbic acid also showed the same inhibition pattern, but at a very low concentration of 1.5 mg/ml, it reached the highest inhibition percentage of 89.8%.

The graph (Figure 1) presented showed that the extract concentrations of both solvents are proportional to the percentage of inhibition, which means the greater the concentration of extracts, the greater the DPPH scavenging activity. The inhibition percentage was significantly greater for the aq. acetone extract of ADF (median = 15.1%) than the aq. ethanol extract of ADF (median = 5.6%) (*p* = 0.011).

### 3.2. Determination of EC_50_ Value for Ajwa Date Flesh Extract with Various Solvents

**Figure 2:**
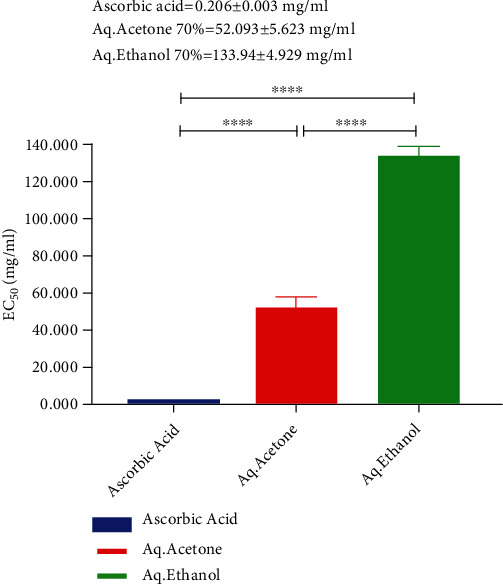
Comparison of EC_50_ value of aq. acetone and aq. ethanol extract of ADF with the standard EC50 value of ascorbic acid. ^∗∗∗∗^Significant difference from control at *p* < 0.05.

EC_50_ is the dose concentration of the sample required to reduce 50% of the free radicals of DPPH. The half-maximal effective concentration (EC_50_) of 70% aq. acetone extract of ADF was 52.09 mg/ml, and EC_50_ of 70% aq. ethanol extract of ADF was 133.94 mg/ml. Ascorbic acid EC_50_ = 0.206 mg/ml. The graphical representation is shown in Figure 2. These results suggest that 70% acetone extract has more antioxidant activity than the 70% ethanol extract of flesh sample. The high EC_50_ value of 70% ethanol extract suggests the minimal amount of antioxidant present in this extract.

### 3.3. Assessment of the Total Antioxidant Contents of the ADP

**Figure 3:**
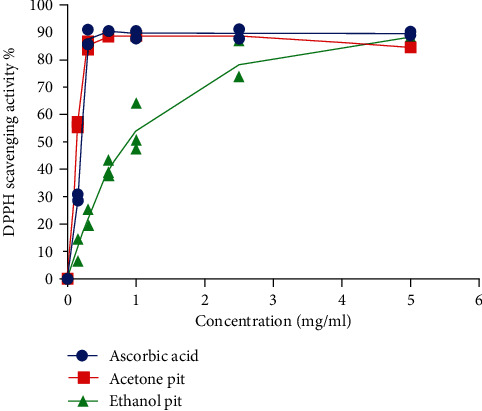
DPPH scavenging activity of various fractions of Ajwa date pits at different concentrations. The curve for ascorbic acid is used as a standard. Each value represents the mean of three independent experiments (*n* = 3).

For ADP sample, concentrations ranging from 0 to 5 mg/ml were selected for both solvents. 70% aqueous acetone extract has greater DPPH free radical scavenging activity at a lower concentration than 70% aqueous ethanol extract of ADP, but the highest average yield of the total antioxidants for both the solvent samples was reached at the concentration of 5 mg/ml as shown in Figure 3. At the *p* < 0.05 level of significance, the results showed that there is significant difference in the inhibition percentage (*p* = 0.04) between the 70% acetone (median = 86.2%) and 70% ethanol (median = 39.1%) solvent extract.

### 3.4. Determination of EC_50_ Value of Ajwa Date Pits with Various Solvents

**Figure 4:**
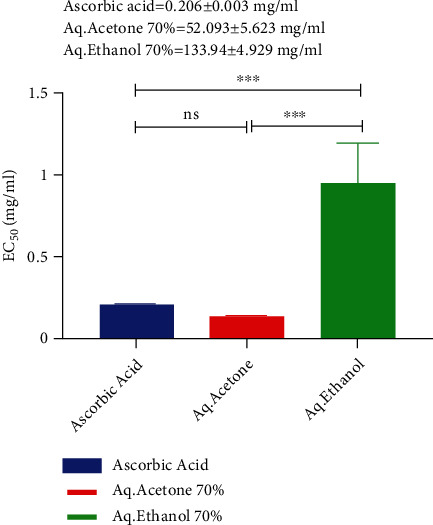
Comparison of EC50 value of aq. acetone and aq. ethanol extract of ADP with the standard EC50 value of ascorbic acid. ^∗∗∗^Significant difference from control at *p* < 0.05; NS: no significant difference from control.

The EC_50_ of 70% aq. acetone extract of ADP was 0.153 mg/ml, and that of 70% aq. ethanol extract of ADP was 0.954 mg/ml. The EC_50_ value of ascorbic acid was 0.206 mg/ml, as shown in Figure 4.

### 3.5. ADF and ADP Induced Changes in Cell Morphology

**Figure 5:**
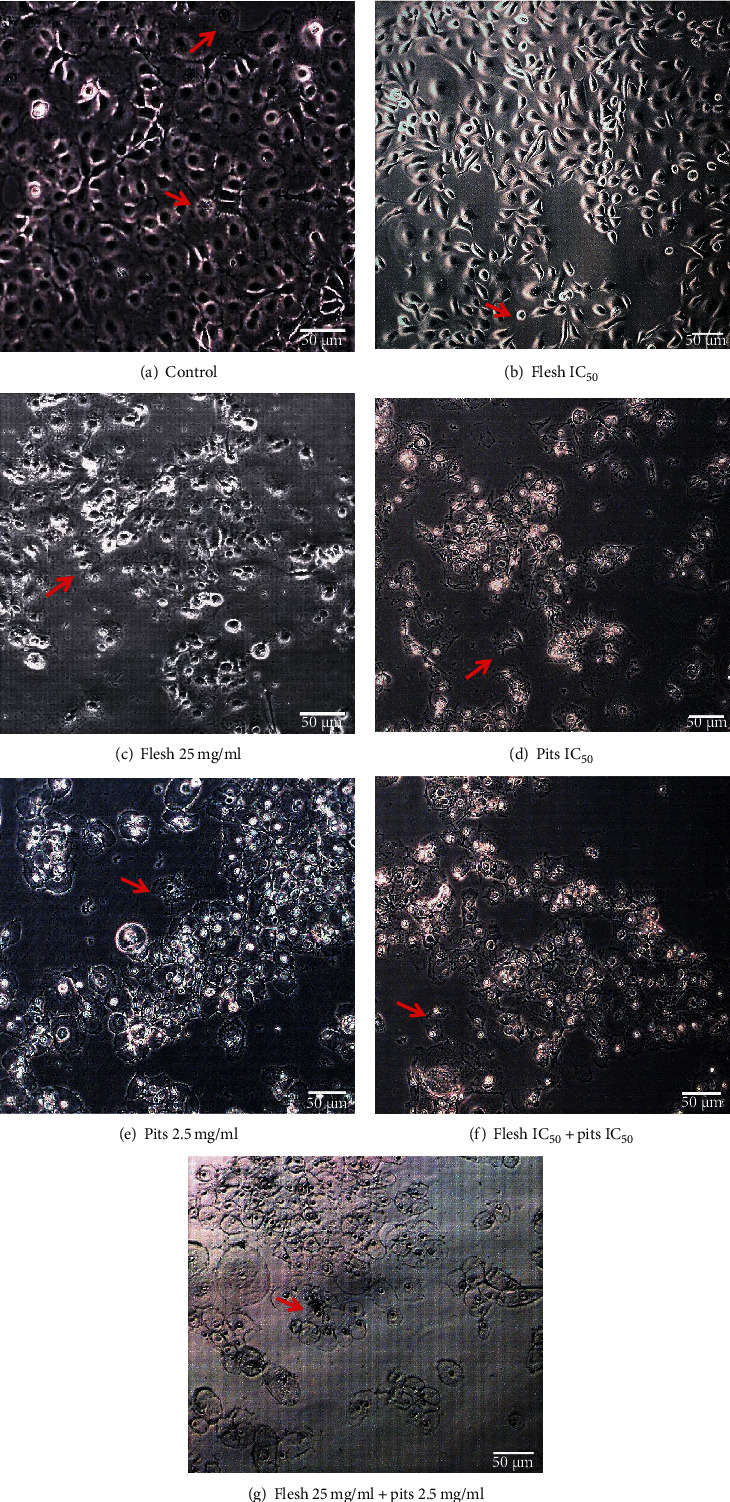
Morphological appearance under an inverted light microscope after 48 hours. Mitotic figures can be seen in (a), cell shrinkage and cell detachment can be seen in (b) and (c), and membrane disruptions, cytoplasmic condensation, and cell death can be seen in (d), (e), (f), and (g); magnification 100x.

Images taken from the light microscope of HSC-2 cells of control sample demonstrated characteristic of epithelial nature and growth proliferation as a monolayer. The cells appeared to be attached together in an ovoid shape with a large central nucleus; dividing cell can also be seen with two or more nucleoli in Figure 5(a). In contrast, the ADF and ADP extract-treated cells showed mild to severe decrease in cell numbers (Figures 5(b)–5(g)) which was dose dependent leaving behind very few live cells. At a concentration of ADF extract IC_50_, cell shrinkage and partial cell-to-cell detachment can be seen; cells have also started losing their shape. At a concentration of ADF extract 25 mg/ml, more drastic changes can be seen; cells have become rounded in shape with complete cell-to-cell detachment, with decrease in number of viable cells. For ADP extract at a concentration of IC_50,_ cytoplasmic vacuolization, nuclear condensation, and cluster shrinkage can be seen. At a concentration of 2.5 mg/ml, a lot of cellular fragmentation can be seen with very few viable cells. For the combination treatment with ADF and ADP extract, drastic morphological changes can be seen in Figures 5(f) and 5(g)); nuclear condensation, cell membrane blebbing, and fragmentation are vastly present with very few viable cells.

### 3.6. ADF Inhibited HCS-2 Cell Proliferation

**Figure 6:**
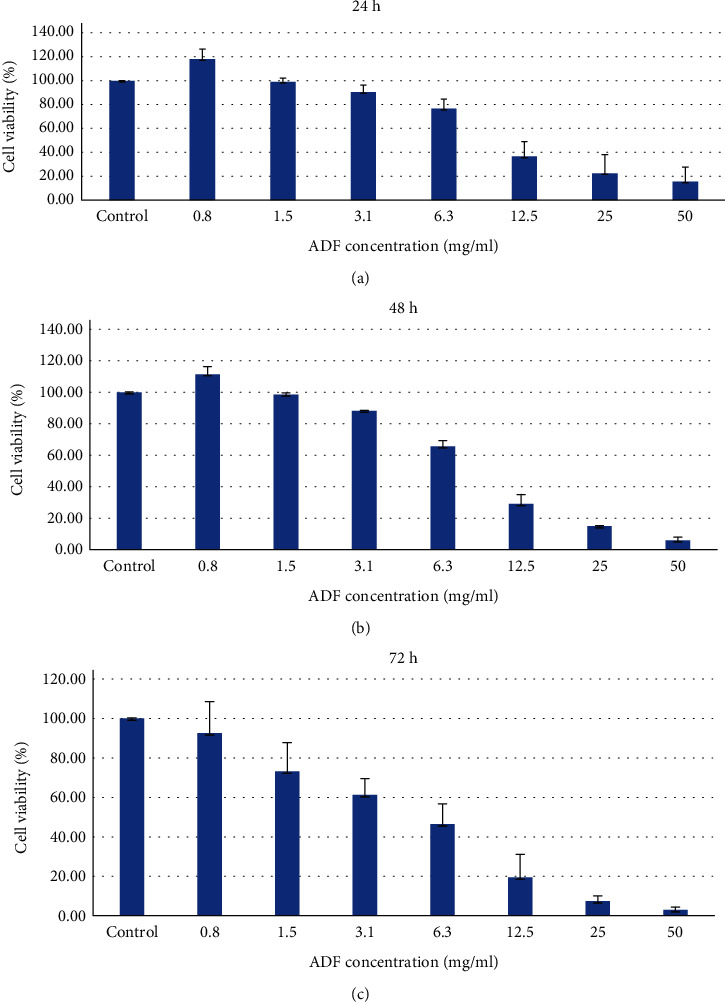
Inhibition of proliferation of HSC-2 cell. MTT assay of HSC-2 cells treated with ADF extract at different concentrations at (a) 24 h, (b) 48 h, and (c) 72 h. These decreases in percentage of cell viability were statistically significant. The values are expressed as mean SD from triplicate samples of three independent experiments. ∗ indicates *p* < 0.05.

**Figure 7:**
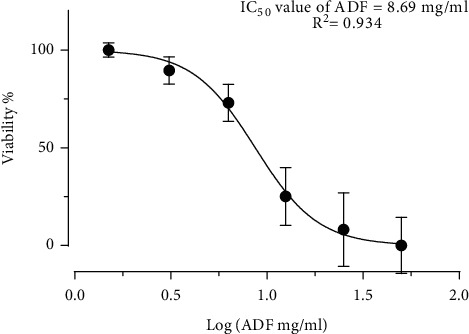
Determination of IC_50_ value of (ADF) on HSC-2 cells.

The MTT assay results from the present study demonstrated HSC-2 cell growth inhibition following the treatment with ADF extract. At 24 h treatment period, ADF extract reduced cell viability to 99, 90.3, 76.5, 36.5, 22.4, and 15.6% at 1.5, 3.1, 6.3, 12.5, 25, and 50 mg/ml, respectively. Negative correlation can be seen between the concentration of ADF extract and viable cells of OSCC. As the concentration of the ADF extract increases from 1.5 to 50 mg/ml, the percentage of viable cells decreased from 99% to 15.6% indicating a dose-dependent manner. At 48 h treatment period, ADF extract exerted a more pronounced effect, drastically reducing the viability of treated cells to 98.5, 88.6, 65.6, 29, 14.8, and 5.9% at 1.5, 3.1, 6.3, 12.5, 25, and 50 mg/ml of extract, respectively. Moreover, a further decrease in cell viability percentage was seen at 72 h with a percentage of 66.5, 61.1 46.3, 19.4, 7.3, and 2.9% at 1.5, 3.1, 6.3, 12.5, 25, and 50 mg/ml, respectively, stipulating that ADF extract is more toxic at higher concentrations, as presented in Figure 6. From the above results at 50 mg/ml concentration, the cell viability decreased to 15.6% at 24 h, 5.9% at 48 h, and 2.9% at 72 h. Therefore, the cell viability data proposed that treatment with ADF extract significantly reduced HSC-2 cell growth in both dose- and time-dependent manners, signifying its ability to impair proliferation potential. IC_50_ value is estimated to comprehend the basic pharmacological and biological characteristics; the lower the IC_50_ value, the more potent the drug is [43]. The IC_50_ value was determined to be 8.69 mg/ml at 24 h duration, in Figure 7.

### 3.7. ADP Inhibited HSC-2 Cell Proliferation

**Figure 8:**
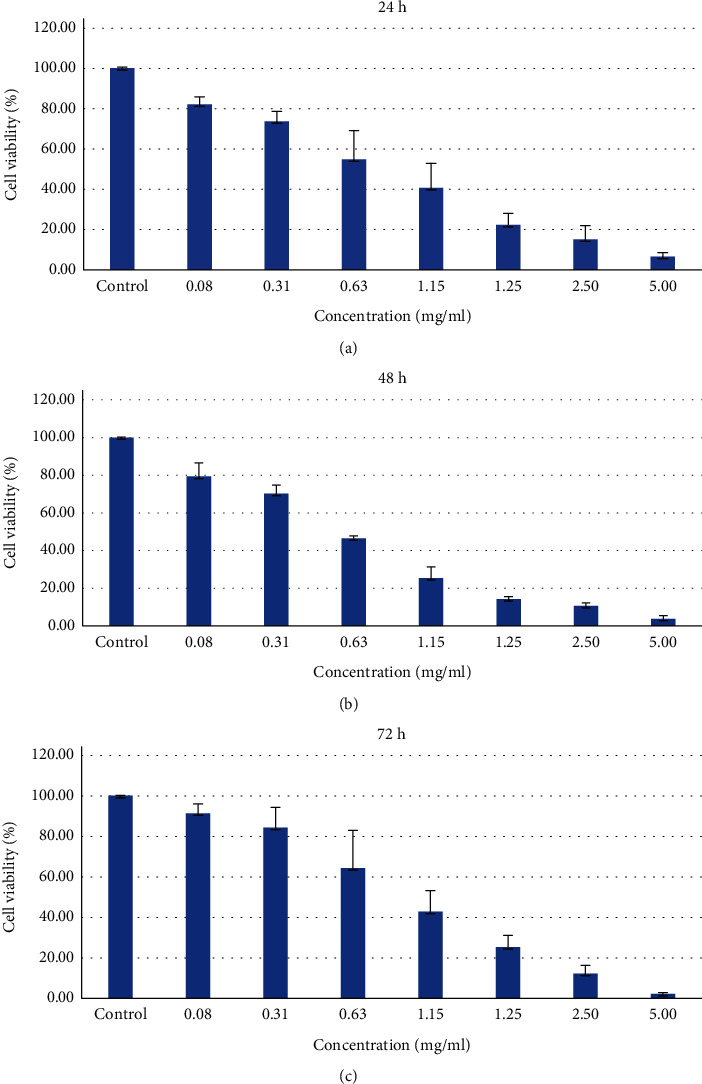
MTT assay of HSC-2 cells treated with ADP extract at different concentrations at (a) 24 h, (b) 48 h, and (c) 72 h. The values are expressed as mean SD from triplicate samples of three independent experiments. ∗ indicates *p* < 0.05.

**Figure 9:**
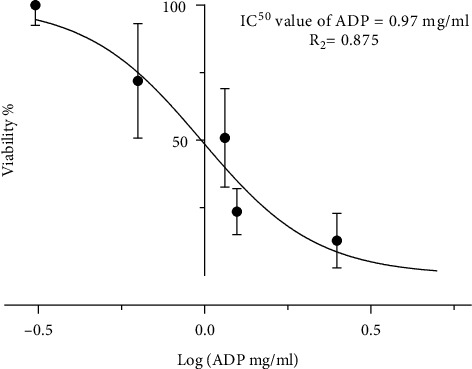
The graph was constructed to determine the IC_50_ value of ADP extract on HSC-2 cells.

MTT assay demonstrated HSC-2 cell growth inhibition following the treatment with ADP extract in a dose-dependent manner (Figure 8). Ajwa date pit extract showed similar but enhanced results compared to the flesh part with significant cytotoxic effect on the viability on HSC-2 cells. At 24 h treatment incubation time, ADP extract reduced cell viability to 82.2, 73.7, 54.9, 40.7, 22.3, 15.1, and 6.6% at 0.08, 0.3, 10.6, 31.1, 51.2, 2.5, and 5.00 mg/ml concentration of ADP extract, respectively. At 48 h treatment period, ADP extract reduced cell viability 79.3, 70.1, 46.5, 25.3, 14.2, 10.6, and 3.7% at 0.08, 0.3, 10.6, 31.1, 51.2, 2.5, and 5.00 mg/ml concentration of ADP extract, respectively, as shown in Figure 8. Thus, the data demonstrated cell growth inhibition in a dose- and time-dependent manner. The significant decrease in percentage of cell viability started from 0.63 mg/ml concentration at 24 h and at 48 hours and 1.25 mg/ml at 72 h. The IC_50_ value was determined to be 0.97 mg/ml at 24 h duration from Figure 9.

### 3.8. ADF and APD Induced Apoptosis in HSC-2 Cells

**Figure 10:**
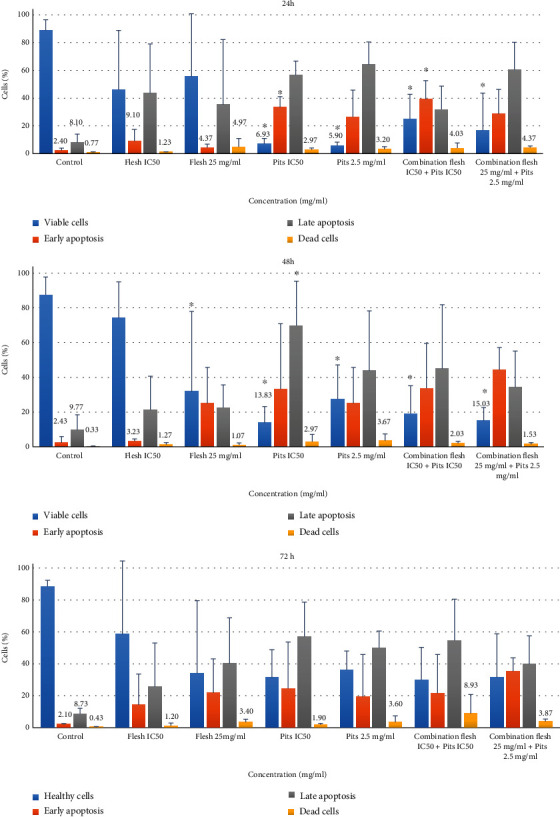
The effect of adding various concentrations of Ajwa date flesh and pit extracts on the apoptotic activity of HSC-2 cell line at 24 h, 48 h, and 72 h. The percentage of cells is shown in four stages, healthy cells, cells in early apoptotic stage, cells in late apoptotic stage, and dead or necrotic cells. The flesh IC50 value is 8.69 mg/ml, and pits IC50 value is 0.97 mg/ml. ∗ indicates that the treatment is significantly different from the control group at *p* < 0.05.

**Figure 11:**
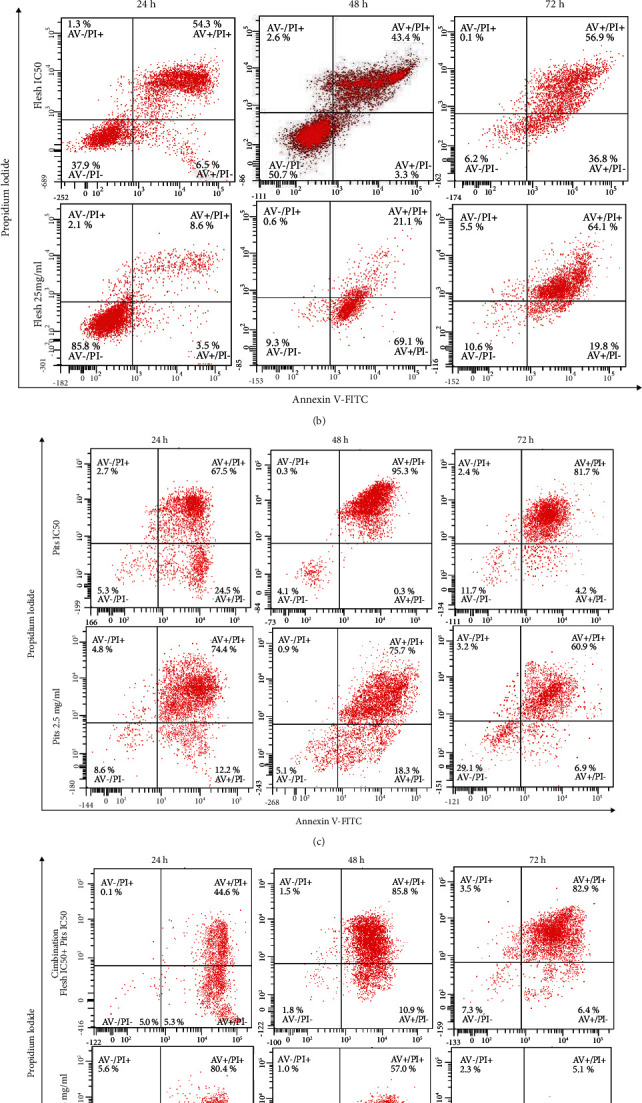
Annexin V-FITC and PI assays on HSC-2 cells treated with various concentrations of ADF and ADP for 24, 48, and 72 hours are represented in a dot-plot graph, where the AV-/PI- quadrant represents viable cells, AV+/PI- quadrant represents cells in early apoptosis, AV+/PI+ quadrant represents cells in late apoptosis, and AV-/PI+ quadrant represents dead or necrotic cells. (a) Represent the control group, (b) represent flesh with IC_50_ value and flesh with 25 mg/ml, (c) shows pits with IC_50_ and 2.5 mg/ml value, and (d) shows the combination of flesh IC_50_+pits IC_50_ and flesh 25 mg/ml+pits 2.5 mg/ml.

The percentage of early apoptotic and late apoptotic cells increased in the treated group when compared to that of the early and late apoptotic cells of control cells. Among the various extracts, ADP 2.5 mg/ml showed the highest percentage of late apoptotic cells and combination of ADF and ADP IC_50_ concentration showed the highest percentage of early apoptotic cells at 24 h. At 48 h, ADP IC_50_ showed the highest percentage of late apoptotic cells and combination of ADF 25 mg/ml+ADP 2.5 mg/ml showed the highest early apoptotic cells. At 72 h, the increase in total apoptotic cells was 40 ± 47.09% and 62.37 ± 50.09% for IC_50_ (8.69) mg/ml and 25 mg/ml of ADF, respectively; for ADP, total apoptotic cells were 82.13 ± 50.7% and 69.77 ± 37.03% for IC50 (0.97) mg/ml and 2.5 mg/ml, respectively, compared to control 10.83 ± 3.87%. (Figures 10 and 11).

When the combination of both ADF and ADP extracts as a single-treatment ADF and ADP IC_50_ (8.69 + 0.97) mg/ml and ADF and ADP (25 + 2.5) mg/ml is given, the result of total apoptotic cells was76.57 ± 50.21 % and 75.47 ± 21.64 % (Figures 10 and 11(a)–11(d)).

## 4. Discussion

The DPPH results of ADF and ADP extracts demonstrated that when acetone was used as a solvent, higher antioxidant activity was obtained in both flesh and pits compared to the values obtained with ethanol at the same solvent concentrations (70%). These results are in line with a previous study conducted by Nematallah et al. in 2018 where 50% aq. acetone yielded the highest antioxidant activity in Ajwa date, followed by ethanolic extract. The variations in the values can be due to the difference in extraction procedure [44]. Another important finding observed from the DPPH assay was that the pit extract exhibited higher antioxidant activity with both the solvents when compared with the flesh part. These results are in support with a study reported by Maqsood *et al.* where the concentration of acetone and ethanol between 60 and 80% yielded the highest DPPH free radical scavenging activity from a date pit [45]. Therefore, it can be concluded that pit appeared to be a richer source for phenols and flavonoids; these results can be supported by previous studies, where date pits are the highest source of total polyphenols among tea, flaxseed, nut seeds, grapes, and even date flesh [46, 47]. Date fruit flesh also contains polyphenols like quercetin and kaempferol [48], which possess anticancer activity against oral squamous cell carcinoma [49, 50]. Therefore, it can be said that the anticancer effect of Ajwa date extract against HSC-2 cells might be due to the integrated or collective effect of the potential bioactive components of Ajwa dates. The anticancer activity of these bioactive compounds can be mediated by several molecular mechanisms including free radical scavenging activity, deactivation of carcinogenic metabolites, antiproliferation, induction of apoptosis, and cell cycle arrest [51].

One of the hallmarks of cancers is its ability to replicate and invade through escaping apoptosis, being insensitive to antigrowth signals, and self-sufficient in growth signals to proliferate uncontrollably. Hence, finding new drugs and treatments, targeting various pathways in the induction of apoptosis and inhibition in the proliferation, plays an important role in the treatment of cancers. Interestingly, the morphological data of the present study revealed ADF- and APD-mediated changes which are indicative of apoptosis. Treated cells appeared shrunken, nonadherent, partially detached, and rounded in shape, with also a decrease in cell number. As the concentration of ADF and ADP extracts increased, more drastic changes were observed like cluster shrinkage, membrane blebbing, and cellular fragmentations leading to cell death, exhibiting a dose-dependent severity. These are typically initial characteristic features of apoptotic cell death [52]. In addition, both MTT and annexin V-FITC costaining with PI assays demonstrated inhibition in cell growth following treatment with ADF and ADP extract, supporting the morphological observations in the HSC-2 cells. In previous studies, apoptosis or initiation of apoptotic pathways has been induced by bioactive components and secondary metabolites of natural products [53].

Interestingly, in the present study, the data from MTT assay showed that, at a concentration of 0.8 mg/ml of ADF extract, there was an increase in cell viability percentage of 117.9 and 111.4% at 24 and 48 h of incubation period, respectively, when compared to the untreated control group. This could be explained by a phenomenon known as hormesis, where some cells might try to adjust to the toxic environment at very low dose resulting in a higher MTT signal compared to the control [54]. ADP extract showed increased cytotoxic effect on the viability on HSC-2 cells, which can mainly be attributed to the presence of high amount of phenolics, flavonoids, and vitamin C in the seeds compared to the flesh part [46]. The date seeds contain high amount of total polyphenols, close to 3942 mg/100 g, whereas date flesh contains 239.5 mg/100 g wet weight [34].

In a study, the IC_50_ value of ADF extract against human hepatocellular carcinoma (HepG2) cells was 20.03 mg/ml after 24-hour exposure [31]. In comparison to the present study, the IC_50_ value of 8.96 mg/ml after 24-hour treatment period indicates that ADF extract was more effective against HSC-2 cells. The variation in the IC_50_ value could be due to many reasons. It could be the difference in proliferation rate of the cells or the cell density during the assay period [55].

On the contrary, the 50% inhibition of HSC-2 cells by ADP extract with an extremely low IC_50_ in the present study confirmed the anticancer property of ADP extract. In a similar study conducted by Thouri and colleagues in 2018, it was shown that date pit extract of two different varieties induced significant growth inhibition and apoptosis in a human cervical cell line (HeLa) and human liver cell line (HepG2) with an IC_50_ value of 0.028 mg/ml and 0.034 mg/ml, respectively. They also reported that the seed extract had no cytotoxic effects on normal fibroblast cell line [41]. Interestingly, polyphenols were distinguished by their low cytotoxic effects towards normal cell line and increased cytotoxicity towards cancer cell line [56].

The exact mode of action of ADF and ADP extract on HSC-2 cells is not fully understood. Nonetheless, a likely route of action for the extract could be via modulating oxidative stress and scavenging free radicals within the cells. Various stimuli including reactive oxygen species are known to activate HSC-2 cells [57]. In the present study, flow cytometry analysis indicated that the cytotoxic effects in HSC-2 cells were due to apoptosis induction. Furthermore, the results also displayed that with a lower concentration of both ADF and ADP extracts, HSC-2 cells were observed more in early apoptosis stage while late apoptosis stage was identified at a higher concentration of extracts. The data also suggests a time-dependent manner. The apoptotic activity exhibited by the ADF and ADP extracts may be attributed to the presence of phenols and flavonoid content like rutin, catechin, caffeic acid, apigenin, and quercetin which are present in Ajwa date flesh and pit [46, 58].

A previous study has reported the induction of apoptosis in breast cancer MCF-7 cells by the methanolic extract of Ajwa date flesh [27], in which it was reported that the percentage of total apoptotic cells was 68.1% at 25 mg/ml at 48 h of treatment duration, which correlates with the findings of present study where the total apoptotic cells at 48 h were 67.1% at 25 mg/ml of ADF extract. With the loss of cell membrane asymmetry, the phosphatidylserine (PS) flips towards the outside, which is considered to be a hallmark of cell in later stages of apoptosis [59]. Furthermore, ADP extract had increased apoptosis, compared to ADF. This result supports the results of MTT assay, in which there was significant cell death after the treatment of Ajwa date extract; apoptosis assay elaborates that the cell went through early and late stages of apoptosis before dying, thus confirming that Ajwa date induced apoptotic cell death in oral cancer cells.

The present study also investigated the combined effect of ADF and ADP extracts for the induction of apoptosis in HSC-2 cells. At 24 h, the percentage of early apoptotic cells with the combination treatment was 39.2% whereas when treated separately, it was 9.10% and 33.4% for ADF and ADP extract, respectively. Although it was not significantly higher than the single treatment, this may suggest that Ajwa date can induce higher level of apoptotic effect when used as a whole. Previously, many studies have evaluated the bioactive phenolic compounds of fruit seed more than the fruit flesh. Similar to the present study, grape seed extract had induced apoptotic cell death in OSCC [60]. Not only the seed but the peel (skin) and the edible part of grape have demonstrated apoptotic activity [61, 62]. Many other fruits have demonstrated higher polyphenol content in their seed than the edible flesh [47]. Therefore, it can be suggested that the fruit as a whole can be more beneficial in providing protection against carcinogenic effects and the seed part can be utilized in many different forms; like recently, date pit powder was used to make noncaffeinated coffee with coffee flavour [63].

## 5. Conclusion

In conclusion, the results from the present study suggest that Ajwa date (flesh and pit) demonstrated significant cytotoxic and antiproliferative activity against HSC-2 cells. The IC_50_ value demonstrated that Ajwa pit had a stronger antiproliferative effect than the flesh extract signifying its higher anticancerous potential, which was further confirmed in morphological alteration such as nuclear shrinkage, blebbing of cell membrane, and reduction in cell number, which are characteristic features of apoptotic cells. Furthermore, ADF and ADP extract was found to cause cell death via apoptosis in OSCC cells by flow cytometry assay in a dose- and time-dependent manner. The present study also demonstrates that Ajwa date as a whole can induce apoptosis more effectively than as separately. As research is still ongoing, further studies can be conducted to purify and identify individual components of the Ajwa date flesh and seed that are responsible for the anticancer properties and to better understand the in-depth molecular mechanism of action of apoptosis so that a novel chemotherapeutic drug can be made with less/no conventional side effects.

## Data Availability

All data are available within the manuscript.

## References

[1] Warnakulasuriya S. (2009). Global epidemiology of oral and oropharyngeal cancer. *Oral Oncology*.

[2] Sung H., Ferlay J., Siegel R. L. (2021). Global cancer statistics 2020: GLOBOCAN estimates of incidence and mortality worldwide for 36 cancers in 185 countries. *CA: a Cancer Journal for Clinicians*.

[3] Kalavrezos N., Scully C. (2015). Mouth cancer for clinicians part 5: risk factors (other). *Dental Update*.

[4] Yakob M., Fuentes L., Wang M. B., Abemayor E., Wong D. T. (2014). Salivary biomarkers for detection of oral squamous cell carcinoma: current state and recent advances. *Current oral health reports*.

[5] Cragg G. M., Grothaus P. G., Newman D. J. (2009). Impact of natural products on developing new anti-cancer agents. *Chemical Reviews*.

[6] Al-Farsi M., Alasalvar C., Morris A., Baron M., Shahidi F. (2005). Comparison of antioxidant activity, anthocyanins, carotenoids, and phenolics of three native fresh and sun-dried date (Phoenix dactylifera L.) varieties grown in Oman. *Journal of Agricultural and Food Chemistry*.

[7] al-Qarawi A. A., Ali B. H., al-Mougy S. A., Mousa H. M. (2003). Gastrointestinal transit in mice treated with various extracts of date ( _Phoenix dactylifera_ L.). *Food and Chemical Toxicology*.

[8] Khalid S., Khalid N., Khan R. S., Ahmed H., Ahmad A. (2017). A review on chemistry and pharmacology of Ajwa date fruit and pit. *Trends in Food Science & Technology*.

[9] Hasan M., Mohieldein A. (2016). In vivo evaluation of anti diabetic, hypolipidemic, antioxidative activities of Saudi date seed extract on streptozotocin induced diabetic rats. *Journal of Clinical and Diagnostic Research: JCDR*.

[10] Umar Ibrahim M., Nordin S., Abdulkareem U., Ibrahim Haruna S., Atif Amin B., Thant Z. (2015). Anti-inflammatory and analgesic activities of aqueous extract date palm (Phoenix dactylifera L) fruit in rats. *Int J Novel Res Healthcare Nurs*.

[11] Ishurd O., Kennedy J. F. (2005). The anti-cancer activity of polysaccharide prepared from Libyan dates (Phoenix dactylifera L.). *Carbohydrate Polymers*.

[12] Assirey E. A. R. (2015). Nutritional composition of fruit of 10 date palm (Phoenix dactyliferaL.) cultivars grown in Saudi Arabia. *Journal of Taibah University for science*.

[13] Bouhlali E. . T., Bammou M., Sellam K., Benlyas M., Alem C., Filali-Zegzouti Y. (2016). Evaluation of antioxidant, antihemolytic and antibacterial potential of six Moroccan date fruit ( _Phoenix dactylifera_ L.) varieties. *Journal of King Saud University-Science*.

[14] Bokhari N. A., Perveen K. (2012). In vitro inhibition potential of Phoenix dactylifera L. extracts on the growth of pathogenic fungi. *Journal of Medicinal Plants Research*.

[15] Jassim S. A., Naji M. A. (2010). In vitro evaluation of the antiviral activity of an extract of date palm (Phoenix dactylifera L.) pits on a Pseudomonas phage. *Evidence-based Complementary and Alternative Medicine*.

[16] Khalid S., Ahmad A., Masud T., Asad M. J., Sandhu M. (2016). Nutritional assessment of ajwa date flesh and pits in comparison to local varieties. *Journal of Plant and Animal Sciences*.

[17] Al-Shahib W., Marshall R. J. (2003). The fruit of the date palm: its possible use as the best food for the future?. *International Journal of Food Sciences and Nutrition*.

[18] Ahmed A., Arshad M. U., Saeed F., Ahmed R. S., Chatha S. A. S. (2016). Nutritional probing and HPLC profiling of roasted date pit powder. *Pakistan Journal of Nutrition*.

[19] Sawaya W. N., Safi W. M., Black L. T., Mashadi A. S., Al Muhammad M. M. (1983). Physical and chemical characterisation of the major date varieties grown in Saudi Arabia, 2: Sugars, tannins, vitamins A and C. *Date Palm Journal*.

[20] Abdul‐Hamid N. A., Abas F., Ismail I. S., Shaari K., Lajis N. H. (2015). Influence of different drying treatments and extraction solvents on the metabolite profile and nitric oxide inhibitory activity of Ajwa dates. *Journal of Food Science*.

[21] Elmaa S. N., Badarushama K., Roslib D., Salvamania S., Hassana M. S., Hashima R. (2018). Solvents extraction effects on bioactive compounds of Ajwa date (Phoenix dactylifera L.) flesh using mixture design. *Chemical Engineering*.

[22] Hong Y. J., Tomas-Barberan F. A., Kader A. A., Mitchell A. E. (2006). The flavonoid glycosides and procyanidin composition of Deglet Noor dates (Phoenix dactylifera). *Journal of Agricultural and Food Chemistry*.

[23] Chaira N., Smaali M. I., Martinez-Tomé M., Mrabet A., Murcia M. A., Ferchichi A. (2009). Simple phenolic composition, flavonoid contents and antioxidant capacities in water-methanol extracts of Tunisian common date cultivars (PhoenixdactyliferaL.). *International journal of food sciences and nutrition*.

[24] Eid N. M., al-Awadi B., Vauzour D., Oruna-Concha M. J., Spencer J. P. E. (2013). Effect of cultivar type and ripening on the polyphenol content of date palm fruit. *Journal of Agricultural and Food Chemistry*.

[25] Ding Y., Ding C., Ye N. (2016). Discovery and development of natural product oridonin-inspired anticancer agents. *European Journal of Medicinal Chemistry*.

[26] Nichenametla S. N., Taruscio T. G., Barney D. L., Exon J. H. (2006). A review of the effects and mechanisms of polyphenolics in cancer. *Critical Reviews in Food Science and Nutrition*.

[27] Khan F., Ahmed F., Pushparaj P. N. (2016). Ajwa date (Phoenix dactylifera L.) extract inhibits human breast adenocarcinoma (MCF7) cells in vitro by inducing apoptosis and cell cycle arrest. *PLoS One*.

[28] Dammak I., Boudaya S., Abdallah F. B., Hamida T., Attia H. (2009). Date seed oil inhibits hydrogen peroxide-induced oxidative stress in normal human epidermal melanocytes. *Connective Tissue Research*.

[29] Ines D., Sonia B., Fatma B. A. (2010). Date seed oil inhibits hydrogen peroxide-induced oxidative stress in human epidermal keratinocytes. *International Journal of Dermatology*.

[30] Kchaou W., Abbès F., Mansour R. B., Blecker C., Attia H., Besbes S. (2016). Phenolic profile, antibacterial and cytotoxic properties of second grade date extract from Tunisian cultivars (Phoenix dactylifera L.). *Food Chemistry*.

[31] Siddiqui S., Ahmad R., Khan M. A., Upadhyay S., Husain I., Srivastava A. N. (2019). Cytostatic and anti-tumor potential of Ajwa date pulp against human hepatocellular carcinoma HepG2 cells. *Scientific Reports*.

[32] Zhang C. R., Aldosari S. A., Vidyasagar P. S., Shukla P., Nair M. G. (2017). Health-benefits of date fruits produced in Saudi Arabia based on in vitro antioxidant, anti-inflammatory and human tumor cell proliferation inhibitory assays. *Journal of the Saudi Society of Agricultural Sciences*.

[33] Dai J., Mumper R. J. (2010). Plant phenolics: extraction, analysis and their antioxidant and anticancer properties. *Molecules*.

[34] Al-Farsi M. A., Lee C. Y. (2008). Nutritional and functional properties of dates: a review. *Critical Reviews in Food Science and Nutrition*.

[35] Ragab A. R., Elkablawy M. A., Sheik B. Y., Baraka H. N. (2012). Antioxidant and tissue-protective studies on Ajwa extract: dates from Al-Madinah Al-Monwarah, Saudia Arabia. *Journal of Environmental & Analytical Toxicology*.

[36] Chan K. W., Khong N. M., Iqbal S., Ismail M. (2013). Isolation and antioxidative properties of phenolics-saponins rich fraction from defatted rice bran. *Journal of Cereal Science*.

[37] Ramasamy S., Wahab N. A., Abidin N. Z., Manickam S. (2011). Cytotoxicity evaluation of five selected Malaysian Phyllanthaceae species on various human cancer cell lines. *Journal of Medicinal Plants Research*.

[38] Brand-Williams W., Cuvelier M.-E., Berset C. (1995). Use of a free radical method to evaluate antioxidant activity. *LWT-Food science and Technology*.

[39] Mirza M. B., Elkady A. I., Al-Attar A. M., Syed F. Q., Mohammed F. A., Hakeem K. R. (2018). Induction of apoptosis and cell cycle arrest by ethyl acetate fraction of Phoenix dactylifera L.(Ajwa dates) in prostate cancer cells. *Journal of Ethnopharmacology*.

[40] Al-Zubaidy N., Al-Zubaidy A., Sahib H. (2016). The anti-proliferative activity of phoenix dactylifera seed extract on MCF-7 breast cancer cell line. *Int J Pharm Sci Rev Res*.

[41] Thouri A., La Barbera L., Canuti L. (2019). Antiproliferative and apoptosis-inducing effect of common Tunisian date seed (var. Korkobbi and Arechti) phytochemical-rich methanolic extract. *Environmental Science and Pollution Research*.

[42] Khan F., Aldhahri M., Hussain M. A. (2018). Encapsulation of 5-flurouracil into PLGA nanofibers and enhanced anticancer effect in combination with Ajwa-dates-extract (Phoenix dactylifera L.). *Journal of Biomedical Nanotechnology*.

[43] He Y., Zhu Q., Chen M. (2016). The changing 50% inhibitory concentration (IC50) of cisplatin: a pilot study on the artifacts of the MTT assay and the precise measurement of density-dependent chemoresistance in ovarian cancer. *Oncotarget*.

[44] Nematallah K. A., Ayoub N. A., Abdelsattar E. (2018). Polyphenols LC-MS2 profile of Ajwa date fruit (Phoenix dactylifera L.) and their microemulsion: potential impact on hepatic fibrosis. *Journal of Functional Foods*.

[45] Maqsood S., Kittiphattanabawon P., Benjakul S., Sumpavapol P., Abushelaibi A. (2015). Antioxidant activity of date (Phoenix dactylifera var. Khalas) seed and its preventive effect on lipid oxidation in model systems. *International Food Research Journal*.

[46] Habib H. M., Platat C., Meudec E., Cheynier V., Ibrahim W. H. (2014). Polyphenolic compounds in date fruit seed (Phoenix dactylifera): characterisation and quantification by using UPLC-DAD-ESI-MS. *Journal of the Science of Food and Agriculture*.

[47] Soong Y.-Y., Barlow P. J. (2004). Antioxidant activity and phenolic content of selected fruit seeds. *Food Chemistry*.

[48] Abdul-Hamid N. A., Mediani A., Maulidiani M. (2018). Metabolite characterization of different palm date varieties and the correlation with their NO inhibitory activity, texture and sweetness. *Journal of Food Science and Technology*.

[49] Zhang C., Hao Y., Sun Y., Liu P. (2019). Quercetin suppresses the tumorigenesis of oral squamous cell carcinoma by regulating microRNA-22/WNT1/*β*-catenin axis. *Journal of Pharmacological Sciences*.

[50] Lin C. W., Chen P. N., Chen M. K. (2013). Kaempferol reduces matrix metalloproteinase-2 expression by down-regulating ERK1/2 and the activator protein-1 signaling pathways in oral cancer cells. *PLoS One*.

[51] Ren W., Qiao Z., Wang H., Zhu L., Zhang L. (2003). Flavonoids: promising anticancer agents. *Medicinal Research Reviews*.

[52] Taatjes D. J., Sobel B. E., Budd R. C. (2008). Morphological and cytochemical determination of cell death by apoptosis. *Histochemistry and Cell Biology*.

[53] Thomasset S. C., Berry D. P., Garcea G., Marczylo T., Steward W. P., Gescher A. J. (2007). Dietary polyphenolic phytochemicals—promising cancer chemopreventive agents in humans? A review of their clinical properties. *International Journal of Cancer*.

[54] Stebbing A. (1982). Hormesis -- The stimulation of growth by low levels of inhibitors. *Science of the Total Environment*.

[55] Thomasset S. C., Berry D. P., Garcea G., Marczylo T., Steward W. P., Gescher A. J. (2017). Measuring cancer drug sensitivity and resistance in cultured cells. *Current protocols in chemical biology*.

[56] Park H. K., Han D. W., Park Y. H., Park J. C. (2005). Differential biological responses of green tea polyphenol in normal cells vs. cancer cells. *Current Applied Physics*.

[57] Razzaghi-Asl N., Garrido J., Khazraei H., Borges F., Firuzi O. (2013). Antioxidant properties of hydroxycinnamic acids: a review of structure-activity relationships. *Current Medicinal Chemistry*.

[58] Saleh E. A., Tawfik M. S., Abu-Tarboush H. M. (2011). Phenolic contents and antioxidant activity of various date palm (&lt;i&gt;Phoenix dactylifera &lt;/i&gt; L.) fruits from Saudi Arabia. *Food and Nutrition Sciences*.

[59] Saraste A., Pulkki K. (2000). Morphologic and biochemical hallmarks of apoptosis. *Cardiovascular Research*.

[60] Aghbali A., Hosseini S. V., Delazar A. (2013). Induction of apoptosis by grape seed extract (Vitis vinifera) in oral squamous cell carcinoma. *Bosnian Journal of Basic Medical Sciences*.

[61] Nirmala J. G., Celsia S. E., Swaminathan A., Narendhirakannan R. T., Chatterjee S. (2018). Cytotoxicity and apoptotic cell death induced by Vitis vinifera peel and seed extracts in A431 skin cancer cells. *Cytotechnology*.

[62] Singha I., Das S. K. (2015). *Grapevine fruit extract protects against radiation-induced oxidative stress and apoptosis in human lymphocyte*.

[63] Baliga M. S., Baliga B. R. V., Kandathil S. M., Bhat H. P., Vayalil P. K. (2011). A review of the chemistry and pharmacology of the date fruits (Phoenix dactylifera L.). *Food Research International*.

